# Cytomegalovirus pp71 Protein Is Expressed in Human Glioblastoma and Promotes Pro-Angiogenic Signaling by Activation of Stem Cell Factor

**DOI:** 10.1371/journal.pone.0068176

**Published:** 2013-07-05

**Authors:** Lisa A. Matlaf, Lualhati E. Harkins, Vladimir Bezrookove, Charles S. Cobbs, Liliana Soroceanu

**Affiliations:** 1 California Pacific Medical Center Research Institute, San Francisco, California, United States of America; 2 Birmingham Veterans Administration Hospital, Birmingham, Alabama, United States of America; 3 University of California San Francisco, Department of Neurological Surgery, San Francisco, California, United States of America; The Ohio State University, United States of America

## Abstract

Glioblastoma multiforme (GBM) is a highly malignant primary central nervous system neoplasm characterized by tumor cell invasion, robust angiogenesis, and a mean survival of 15 months. Human cytomegalovirus (HCMV) infection is present in >90% of GBMs, although the role the virus plays in GBM pathogenesis is unclear. We report here that HCMV pp71, a viral protein previously shown to promote cell cycle progression, is present in a majority of human GBMs and is preferentially expressed in the CD133+, cancer stem-like cell population. Overexpression of pp71 in adult neural precursor cells resulted in potent induction of stem cell factor (SCF), an important pro-angiogenic factor in GBM. Using double immunofluorescence, we demonstrate in situ co-localization of pp71 and SCF in clinical GBM specimens. pp71 overexpression in both normal and transformed glial cells increased SCF secretion and this effect was specific, since siRNA mediated knockdown of pp71 or treatment with the antiviral drug cidofovir resulted in decreased expression and secretion of SCF by HCMV-infected cells. pp71- induced upregulation of SCF resulted in downstream activation of its putative endothelial cell receptor, c-kit, and angiogenesis as measured by increased capillary tube formation *in vitro*. We demonstrate that pp71 induces a pro-inflammatory response via activation of NFΚB signaling which drives SCF expression. Furthermore, we show that pp71 levels and NFKB activation are selectively augmented in the mesenchymal subtype of human GBMs, characterized by worst patient outcome, suggesting that HCMV pp71-induced paracrine signaling may contribute to the aggressive phenotype of this human malignancy.

## Introduction

Stem cell factor (also known as SCF, kit-ligand, or steel factor) is a cytokine that binds to the c-kit receptor tyrosine kinase (CD117) and is involved in hematopoesis, spermatogenesis, and melanogenesis. The c-kit receptor, a proto-oncogene activated in several human tumors, is expressed on hematopoetic stem cells, germ cells, and progenitor cells derived from the neural crest [1]. SCF, which exists in both a membrane-bound and a secreted form, is produced by fibroblasts and endothelial cells and promotes cell survival, proliferation, and differentiation by activating multiple signaling cascades downstream of c-kit, including the RAS/ERK, PI3-kinase, Src kinase, and Jak/STAT pathways [1]. Importantly, SCF/c-kit activation has been shown to promote recruitment of endothelial progenitor cells to stimulate angiogenesis in ischemic environments, a process which is essential to the growth and maintenance of tumors [2].

Malignant gliomas are an extremely aggressive type of brain tumor, which are highly vascular and invasive. Because tumor-induced angiogenesis is a pathological hallmark of this malignancy, it is believed that anti-angiogenic strategies have a great potential in improving the treatment and survival of glioma patients [3], [4]. SCF is abundantly expressed in high-grade gliomas and in neurons following traumatic brain injury, and plays an important role in tumor- and host-induced angiogenesis [5].

Human cytomegalovirus (HCMV) is a member of the betaherpesvirus subfamily that has been found to be associated with several human malignancies, including glioblastoma [6], [7], [8], [9], [10], [11], [12], [13], [14], [15], [16], [17]. HCMV, like all other herpesviruses, has the ability to remain latent within the body for the lifetime of the host and can contribute to chronic inflammation, immunosuppression, and metabolic disruption of associated tissues. Several lines of evidence support the idea that HCMV may act as a promoter of the neoplastic process in glioma by modulating the survival, invasive potential, angiogenic properties, chromosome stability, DNA damage response, immune evasion, and cell cycle control of associated cells [18]. To date, several HCMV proteins have been identified in glioma that display oncogenic features. For example, the viral chemokine receptor US28 can promote a pro-angiogenic and proliferative phenotype through IL6-Stat3 signaling activation and the viral glycoprotein gB can bind and activate the cellular PDGFRα receptor, which drives proliferation and tumorigenesis of glial precursors through persistent PI3K-Akt activation [19], [20], [21].

The HCMV tegument protein pp71 performs many functions to enhance the efficiency of viral gene expression and replication. At the start of infection, pp71 stimulates viral immediate early gene expression by degrading the cellular repressor protein Daxx [22], [23]. Degradation of Daxx in conjunction with sumoylation of PML by the viral protein IE1 results in the dispersal and disregulation of associated cellular regulatory proteins during infection [24]. pp71 has also been demonstrated to degrade the hypophosphorylated form of the retinoblastoma (Rb) tumor suppressor protein in fibroblasts, thus promoting cell cycle progression into S phase [25], [26]. In addition, pp71 downregulates MHC class I cell surface expression in glioblastoma cells, thus facilitating immune evasion [27]. Based on these proposed activities of pp71, we hypothesized that the pp71 protein may mediate additional oncomodulatory effects of HCMV in human GBMs. Here, we investigate the role of HCMV pp71 in modulating SCF and NFKB signaling, as they relate to glioblastoma pathogenesis.

## Materials and Methods

### Ethics Statement

All human brain tissues (including glioblastoma samples processed as described below) used in these studies were obtained from the CPMC Neurosurgery Department, under an IRB approved protocol (Protocol # 25.125-1). All patients provided written consent stating that they allowed for their tumor samples to be used for basic research. The California Pacific Medical Center Institutional Review Board Panel#1 approved the tissue collection protocol, including the patient consent forms (Current IRB Assurance NO: FWA00000921). Samples have been de-identified before being processed, to protect patient privacy. NPC cells were a gift from Dr. Dennis Steindler (please see reference 28 for details about how the line was produced).

### Cell Culture

U87 human glioblastoma cells were obtained from ATCC and grown in DMEM/F12+10% FBS. The NPC cell line was characterized by immunofluorescence and found positive for Nestin, GFAP, Tuj1, and Olig 2. NPCs were grown in DMEM-F12+5% FBS+Bovine Pituitary Extract (Invitrogen)+N2 supplement (Invitrogen) +20 ng/ml EGF +20 ng/ml bFGF. HUVEC cells were obtained from Invitrogen and were cultured in Medium 200PRF with or without low serum growth supplement (LSGS) (Invitrogen) according to the manufacturer’s protocol, and were used at passages 1–3. Normal human astrocytes (Lot #0000199740) were obtained from Lonza and grown in astrocyte basal medium supplemented with SingleQuots growth factors, cytokines, and supplements. Primary glioblastoma derived cultures were processed as follows: tissues were dissociated using papain and single cell suspensions were cultured using neural basal medium+ N2 supplement, 20 ng/ml EGF, 20 ng/ml bFGF, and 1 ug/ml laminin (Sigma). GBM cell lines 3832 and 387 were obtained from Dr. Jeremy Rich (Cleveland Clinic) and grown in complete neurobasal medium [28]. Where indicated, CD133+ cells were isolated using the MACS CD133 Microbead kit and an autoMACS magnetic separator (Miltenyi). For ELISA, HUVEC activation, transwell migration, and tube formation assay supernatant collection, cells were cultured in the absence of serum and growth factors for at least 48 hours prior to media collection.

### RNA and Protein Isolation from Whole Tissue

Brain tissue was homogenized and lysed in 1 mL QIAzol reagent (Qiagen) using a TissueRuptor homogenizer (Qiagen). RNA was then chloroform extracted and purified using the RNeasy lipid tissue mini kit (Qiagen). The quality of the RNA was verified by spectrometry and visualization of ribosomal RNA bands on an agarose gel. DNA was precipitated from the remaining interphase and organic phase with 75% ethanol, and the protein in the supernatant was then isopropanol precipitated, denatured with 0.3 M guanidine hydrochloride, and resuspended in 1% SDS. Protein fractionation was performed on cells homogenized in cold PBS from frozen tissue using a subcellular protein fractionation kit (Pierce) according to the manufacturer’s instructions.

### PCR Analysis

For standard end-point PCR experiments, 1 ug of each RNA was reverse transcribed into cDNA using the iScript cDNA synthesis kit (BioRad) or the SuperScript II kit (Invitrogen) and PCR amplified using the Taq PCR core kit (Qiagen) with an input of 50 ng of cDNA for each experimental sample and water only for the negative control. Control RNA from fetal brain and adult normal cortex were commercially obtained (BioChain). The primers used for PCR analysis are as follows: pp71F 5′-AGAAACACGCTGGTCGGCGG-3′, R 5′-CGCGGCGGCGAAGAAAATCG-3′, Rab14 F-GCAGATTTGGGATACAGCAGG-3′, R-5′-CAGTGTTTGGATTGGTGAGATTC. All PCR reactions were performed in 50 uL volumes with 50 amplification cycles, and an annealing temperature of 60 degrees for the pp71 primers and 58 degrees for the Rab14 primers. 20 uL of each PCR reaction was resolved on a 1% agarose gel and the size of each amplicon (pp71 = 390 base pairs, Rab14 = 167 base pairs) was verified relative to a 1 KB DNA ladder (Fermentas). The DNA from the remainder of each PCR reaction was then isolated using the MinElute PCR Purification kit (Qiagen) and sequenced. TaqMan analysis was performed using custom pp71 and US28 primers and probes (pp71R-5′-TCAGGCCGTTCATTTGGAA-3′, pp71R-5′AACCCACGGCGGAAAAAG-3′, FAM-CCGACAGCCGCTAGGCCGC-TAMRA, US28F-5′- CGGCAACTTCTTGGTGATCTTC-3′, US28R-5′- CATCGCCGGAGCATTGA-3′, FAM- CCATCACCTGGCGACGTCGGA-TAMRA) and SCF/KITLG, CXCL12, IL8, c-myb Rab14, GAPDH, Sox11, PDGFRα, Olig2, Twist, CHI3L1, and CEBPB primer/probe mixes were purchased from Applied Biosystems. All assays were performed with Applied Biosystems TaqMan FAST Universal PCR Master Mix and the 7500 fast real-time PCR system. When necessary, standard curves were generated with serial dilutions of Ad169 viral DNA (Advanced Biotechnologies) or human genomic DNA (Applied Biosystems). Cytokines RANKL (40 ng/mL) and TNFα (20 ng/mL) were obtained from R&D systems, and the drug Bay11-7085 (5 uM) was obtained from Santa Cruz Biotechnology. Drug and cytokine treatments were performed in serum-free conditions for 24 hours. For quantitative analysis of gene expression using the oncogenes and tumor suppressor genes RT^2^ Profiler PCR array (SA Biosciences), RNA was extracted from adenovirus transduced NPCs using the RNEasy kit (Qiagen) and 1 ug RNA was reverse transcribed into cDNA using the RT^2^ first strand kit (SA Biosciences) according to the manufacturer’s instructions. Resulting cDNA was then mixed with the RT^2^ Sybr Green/ROX master mix and water, and 25 uL of the experimental cocktail was added to each PCR well. Real-time PCR was then performed using an Applied Biosystems 7500 Fast thermocycler according to the manufacturer’s instructions, and data was analyzed using the SA Biosystems online RT^2^ Profiler PCR Array Data Analysis software.

### Serology

Cytomegalovirus IgG and IgM ELISA kits were used for determination of serostatus from patient serum according to the manufacturer’s instructions (IBL International).

### Viruses

Recombinant adenovirus expressing HA-tagged pp71 (rAD-pp71) was kindly provided by Dr. Robert Kalejta (University of Wisconsin, Madison) and has been previously described [25]. The control adenovirus expressing GFP was obtained from Vector Biolabs. All infections were done at 10,000 particles per cell for 48 hours and in the absence of serum where indicated. Retroviruses pLXSN and pLSNX-pp71 were transfected into PT67 packaging cell lines and supernatants were harvested at 32 degrees and used to transduce U87 cells with 6 ug/mL polybrene. U87 cells were selected with 500 ug/mL G418 for 1 week and then G418 was removed. All HCMV infections were performed at an MOI of 3 with either the laboratory strain Towne (ATCC) or the clinical strain TR (gift from Dr. Lee Fortunato, University of Idaho). Presence of the clinical cassette region UL b’ in TR was confirmed by PCR analysis after propagation.

### Antibodies

Mouse antibodies to pp71 have been previously described [29] and were kindly provided by Dr. Tom Shenk (Princeton University). Antibody 2H10-9 was used for western blot at a 1∶10 dilution and 10G11 was used for immunofluorescence at a 1∶2 dilution. The following antibodies were obtained from commercial sources: anti-actin, anti-HA (Sigma), anti-SCF (Epitomics), anti-cKit, anti-phospho-cKit, anti-p38, anti-phospho-p38, anti-Cox2 (Cell Signaling), anti-CD31, anti-p100/p52 (Abcam), anti-RelB, anti-p65/RelA, anti-NIK (Santa Cruz Biotechnology), anti-IE1 (mAB810), anti-Olig2, anti-CD44 (Millipore) and anti-RB (BD Biosciences).

### ELISA

Supernatants from cultured cells were subjected to ELISA using the DuoSet human SCF kit DY255 (R&D Systems) according to the manufacturer’s instructions.

### RNA Interference

Knockdown of pp71 was achieved with 2 custom siRNA oligonucleotide duplex designed and synthesized by Dharmacon. The sense sequences for the siRNAs are: A = GCATACATCCCGAGTACATTT, B = AGGAAGAGGACGACGAAGAUU. A non-targeting control pool was used as a negative control (Dharmacon D-001810-10-05). Transfections were performed for 72 hours according to Dharmacon’s suggested protocol with Lipofectamine 2000 (Invitrogen) transfection agent. For U87 cells the optimal amount of Lipofectamine 2000 was used (10 uL per well in a 6-well dish). Half this amount was used for primary cultures to alleviate toxicity. For RelB knockdown, a pool of 3 siRNAs specific to RelB was used (sc-36402) according to the manufacturer’s instructions (Santa Cruz Biotechnology).

### Immunostaining

Cells were grown on glass slides in 24-well tissue culture dishes and treated as indicated. Prior to staining, cells were rinsed with PBS, fixed for 10 minutes in cold methanol, and then blocked for 30 minutes with protein free blocking buffer (Thermo Fisher). Primary antibodies were used as indicated, and the following secondary antibodies were used at a dilution of 1∶1000: AlexaFluor488 anti-mouse IgG, AlexaFluor647 anti-rabbit IgG, and AlexaFluor647 anti-goat IgG (Invitrogen). Cells and were visualized using a Nikon Eclipse C1 Confocal microscope (Nikon TE2000-U) fitted with a “Cool Snap” Photometrix camera (Roper Scientific). Images were acquired using EZ-C1 v2.20 software and further processed using Adobe Photoshop CS4. Frozen tissue sections were prepared and stained as previously described with modifications [21]. Primary GBM tissues examined using double immunofluorescence are included in Table 1. Briefly, after primary antibody incubation mouse or rabbit fluorescent enhancement probes were applied for 30 minutes (Biocare Medical) followed by incubation with goat anti-rabbit dylight 594 or goat anti-mouse dylight 488 antibodies (Biocare Medical). Images were taken with an Axio Image Z2 microscope (Zeiss). Pearson coefficients were calculated with ImageJ software for pp71 and SCF together (n = 7 cells), pp71 alone (n = 8 cells) and SCF alone (n = 5 cells).

**Table 1:** pp71 status, HCMV serology and IF analysis of primary GBM samples.

	Subclass	RT-PCR	TaqMan	Western	IgG	IgM	pp71 IF	SCF IF	CD31 IF
**CPMC-074**	BC	−	+	N.D.	N/A	N/A	N.D.	N.D.	N.D.
**CPMC-080**	LGA	−	−	−	N/A	N/A	N.D.	N.D.	N.D.
**CPMC-087**	LGA	+	+	−	N/A	N/A	−	−	−
**CPMC-076**	PN	−	+	+	+	−	−	−	+
**CPMC-092**	MES	+	+	+	+	−	+	+	+
**CPMC-047**	MES	+	+	+	−	+	+	+	+
**CPMC-050**	CLAS	+	+	+	+	−	+	+	+
**CPMC-085**	MES	+	+	+	−	+	+	+	+
**CPMC-086**	MES	−	+	N.D.	N/A	N/A	N.D.	N.D.	N.D.
**CPMC-093**	MES	−	+	+	+	−	+	+	−
**CPMC-098**	CLAS	+	+	+	N/A	N/A	N.D.	N.D.	N.D.
**CPMC-102**	MES	+	+	+	+	−	N.D.	N.D.	N.D.
**CPMC-101**	PN	+	+	+	+	−	N.D.	N.D.	N.D.

**Figure 1:**
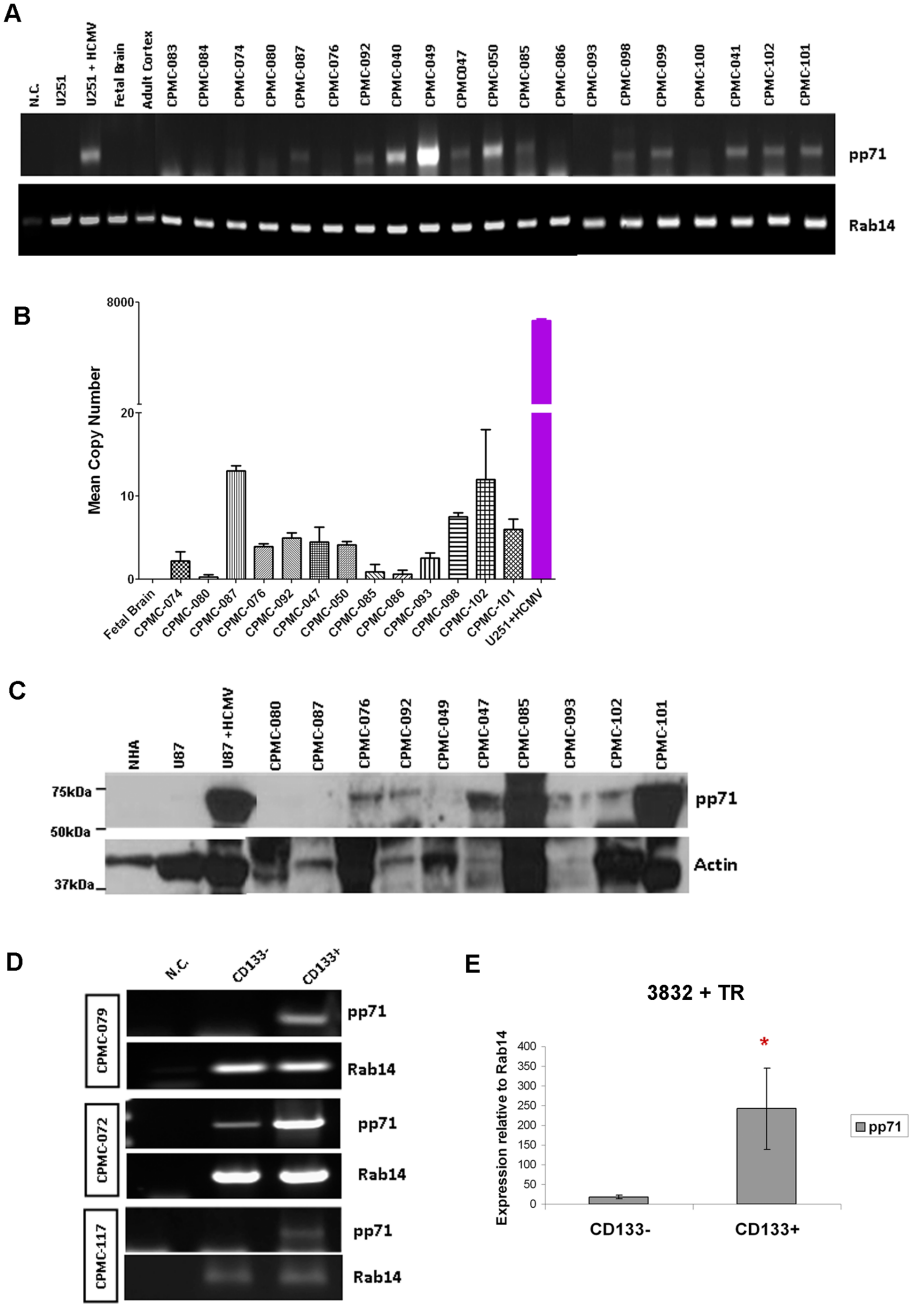
Detection of pp71 RNA and protein in primary glioma specimens. **A:** RNA was extracted from 20 different primary brain tissues, synthesized into cDNA, and amplified using pp71 and Rab14-specific PCR primers. RNA from U251 glioma cells mock infected or infected with HCMV Towne strain and commercially available RNA samples from normal fetal and adult human brain were used as controls. The N.C. negative control PCR contained water in place of cDNA. **B**: Several cDNA samples described in A were analyzed by TaqMan using primers and probes specific for the pp71 gene and normalized to Rab14. Copy number was determined using Ad169 viral DNA standard curve. **C**: Western blot analysis for pp71 from 10 different primary brain tissues. Lysates from normal human astrocytes and mock-infected or HCMV-infected U87 glioma cells were used as controls. **D**: Cells from 3 freshly resected GBM were sorted using CD133 labeled antibody and analyzed by RT-PCR for pp71 and Rab14. Negative control (NC) is PCR performed with water instead of cDNA. **E**: HCMV-infected primary GBM cell line (3832) was sorted using CD133 labeled antibody at 72 hours post-infection and analyzed by TaqMan for pp71 compared to a mock-treated control. The relative expression of pp71 normalized to Rab14 is displayed for 3 independent experiments, *p<0.05 by student t-test.

Serology for IgG and IgM was performed and charted relative to pp71 expression status as determined by RT-PCR, TaqMan and western blot in figure 1. Flash frozen tissue sections were analyzed for the presence of pp71, SCF and CD31 by immunofluorescence (IF) in selected tumor cases, as indicated. GBM patient samples without available plasma were labeled N/A. Tumors were classified as low grade astrocytoma (LGA) or GBM by pathology, and the GBM samples were further subclassified as proneural (PN), mesenchymal (MES), or classical (CLAS) based on their gene expression signatures (data not shown and figure 6C). BC refers to a non-tumor surgical specimen from a blood clot. ND signifies not determined.

### BrdU Incorporation Assay

NPCs were treated as indicated and then BrdU labeled using the 5-Bromo-2′-deoxy-uridine labeling and detection kit III (Roche). Cells were incubated in the labeling reagent for 1 hour and then fixed for 10 minutes in cold methanol. Cells were then stained according to the manufacturer’s protocol, visualized by fluorescence microscopy and the number of BrdU positive cells was calculated and plotted.

### HUVEC Tube Formation Assay

Fresh supernatants from NPCs or U87 cells were harvested, and 20 ng/mL recombinant human SCF (R&D Systems) was incubated with untreated supernatant and 1 ug/mL of anti-SCF neutralizing antibody (R&D Systems) was incubated with rAD-pp71 supernatant for 1 hour at 37 degrees. 150 uL of GelTrex reduced growth factor gel matrix (Invitrogen) was added to the wells of a 24-well culture dish and allowed to solidify at 37 degrees for 30 minutes. HUVEC cells were detached using EDTA and 20,000 cells were added to 250 uL of indicated conditioned medium per well and added to the geltrex containing wells. Tubes were allowed to form 16 hours at 37 degrees and cells were visualized using a Nikon Inverted Eclipse TE-2000E microscope, fitted with a CCD Cascade II camera. NIS Elements AR3.0 was used to acquire images, which were further processed in Photoshop.

### Transwell Migration Assay

6.5 mm polycarbonate transwell permeable supports with an 8 um pore size were used with 24-well tissue culture dishes (Corning). The bottom of the transwell chambers were coated with 5 ug/mL vitronectin overnight at 4 degrees, washed in PBS, then blocked for 1 hour at room temperature in 1% filter-sterilized BSA in serum free medium. 200 uL of conditioned medium from U87 cells or U87 cells treated as indicated were placed in the bottom chamber of the transwell dish and 20,000 HUVEC cells in 0.1% BSA/serum free medium were added to the upper chamber. Dishes were incubated at 37 degrees for 4 hours and the cells that migrated to the bottom side of the chamber were rinsed, fixed in formaldehyde for 10 minutes, and then stained with crystal violet. Stained cells were visualized using a Nikon Inverted Eclipse TE-2000E microscope, fitted with a CCD Cascade II camera. NIS Elements AR3.0 was used to acquire images.

### Microarray Analysis

Affymetrix Human Gene 1.0 ST arrays were processed and anlalyzed as previously published [21]. The raw and RMA normalized microarray data were deposited in the Gene Expression Omnibus database (accession number http://www.ncbi.nlm.nih.gov/geo/query/acc.cgi?token=hxutbkksiwaschc&acc=GSE42618).

### Luciferase Reporter Assay

5000 U87 cells were seeded in white 24-well plates and transfected the next day with a luciferase reporter construct driven either by a scrambled promoter (R01 negative control), a RPL13 promoter (positive control) or the SCF promoter (KITLG-PROM light switch promoter) at a 1∶3 DNA to FuGENE HD transfection reagent (Switch Gear Genomics). Cells were treated as indicated (72 h HCMV infection, 48 hour adenovirus infection or knockdown, and 24 hour cytokine or drug treatment) and then the LightSwitch assay reagent was added for 30 minutes and plates were read with a luminescent plate reader. Each condition was performed in triplicate for each experiment.

## Results

To determine if HCMV pp71 gene products are expressed in human glioblastoma cells, we performed a RT-PCR based screening of RNA obtained from fresh frozen primary brain tissue specimens. We determined that 10/15 GBM specimens had detectable pp71 gene expression, while none of the normal brain tissues (CPMC-083, 084, and 074) and 1/2 samples from low grade astrocytomas (LGA, CPMC-080 and 087) were positive (figure 1A). RT-PCR for the Rab14 gene product, which is a cellular gene that has very little expression variability in GBMs served as a control (figure 1A). The identity of the PCR products in GBM samples was confirmed by sequence analysis, and comparison to HCMV- infected U251 cells provided confirmation that amplified sequences were not laboratory contaminants (figure S1A). For a more sensitive approach to detect low copy numbers of pp71, we performed TaqMan analysis on a subset of samples and were able to detect various levels of pp71 expression in 14 specimens with greater specificity (figure 1B). Three tumors were negative for pp71 by TaqMan (CPMC-41, -080, and -113) and were not plotted. Due to the low level of expression of viral genes in these samples, we tested the effects that the cDNA synthesis reaction (iScript versus SuperScript II) as well as cDNA dilution had on detection of pp71 versus GAPDH in two HCMV-positive cases (figure S1B). The quantity of transcripts detected varied in each condition, indicating that factors other than transcript abundance can interfere with absolute quantification.

We then performed western blot analysis on protein lysates extracted from fresh primary brain specimens to confirm the presence of the pp71 protein in GBMs. A representative blot is shown in Figure 1C, demonstrating pp71 expression in several primary brain samples (7/10) and the positive control HCMV-infected U87 glioma cells, but not in the negative control cell lysates from normal human astrocytes and uninfected U87 cells. Equivalent volumes of each lysate were loaded since quantification of primary protein lysates is often inaccurate due to necrotic cellular debris; therefore the blot was reprobed for the cellular gene actin to account for loading differences. Table 1 summarizes results showing pp71 expression for several primary samples analyzed and corresponding patient HCMV serology status. Due to the variable sensitivity of these different assays few cases of discordance in pp71 detection were noted; however 8 cases were positive by both TaqMan and western blot and were also HCMV seropositive (table 1). Furthermore TaqMan analysis (more sensitive than RT-PCR) displayed a good correlation with protein expression in the same samples.

Our laboratory has found evidence that HCMV gene products are preferentially expressed in the GBM cancer stem cell subpopulation that are often characterized by expression of the CD133 cell surface marker [30]. Therefore we performed RT-PCR on the CD133 positive and negative subpopulations sorted from three fresh, primary GBM samples. As shown in figure 1D, we observed that pp71 RNA was more highly expressed in the CD133 positive subpopulation compared the CD133 negative fraction. To verify this finding, we performed pp71-specific TaqMan on cDNA from an HCMV negative primary GBM cell line (3832) infected with a clinical HCMV strain (TR) in culture, followed by CD133 sorting (figure 1E). We observed a 13-fold higher expression of pp71 in the CD133+ fraction compared to the CD133- negative fraction when normalized to Rab14. These data indicate that expression of pp71 may occur preferentially in cells with cancer stem-like characteristics.

**Figure 2:**
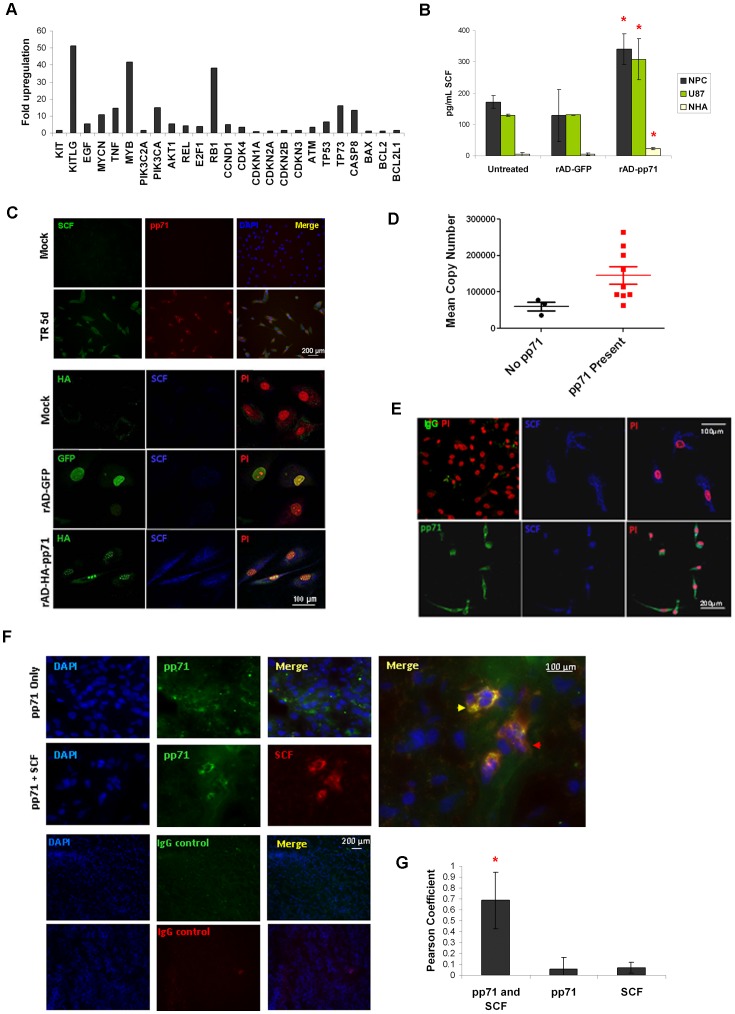
pp71 stimulates expression and secretion of the pro-angiogenic cytokine SCF. **A**: NPC cultures were transduced with adenoviruses expressing either pp71 or GFP as a negative control and total RNA was harvested, reverse transcribed into cDNA, and analyzed using a quantitative PCR array. Fold-change in expression of 24 out of 87 cellular genes in pp71-transduced cells is displayed **B**: Conditioned medium from serum-starved NPCs, U87 cells, or normal human astrocytes treated as indicated were analyzed by ELISA for human SCF. *p<0.05 by student t-test when compared to untreated control; when compared to rAD-GFP control p = 0.037 for NHAs, 0.09 for NPCs, and 0.094 for U87s. **C**: Top panel: NPCs mock-infected or infected with HCMV TR strain for 5 days were immunostained for pp71 (red), SCF (green) and counterstained with DAPI (blue). Bottom panel: NPCs mock-infected or transduced with rAD-GFP or rAD-pp71 were immunostained for the pp71 associated HA tag (green – mock and rAD-pp71 only), human SCF (blue) and counterstained propidium iodide (red). **D**: Several cDNA samples as described in figure 1A were analyzed by TaqMan for SCF expression. Copy number was determined using a human brain cDNA standard curve and is plotted relative to the presence of pp71 transcripts in the same specimens. **E**: Primary GBM cells were immunostained for pp71 (green) and SCF (blue). Secondary antibody only was a negative control (IgG-green) and counterstain was performed with propidium iodide (red). **F**: Frozen tissue sections from primary GBMs were processed by immunostaining for pp71 only (green) or pp71 and SCF (red, red arrow). Areas of co-localization in the merged image are yellow (yellow arrow). Tissues were counterstained with DAPI. Controls for immunofluorescent staining of frozen tissue sections were performed with secondary antibody only (anti-mouse IgG 488 and anti-rabbit IgG 594). **G**: The Pearson coefficient for green (pp71) and red (SCF) pixels were calculated in both co-stained and single-stained samples from 7 cells and plotted (quantification was performed as described in reference ^21^). pp71 and SCF staining was highly correlated (r = 0.69) and significantly different from single stained controls (*, p = 0.001 for pp71 and 0.002 for SCF).

Given the propensity for pp71 expression in glioma stem like cells, we hypothesized that HCMV pp71 expression may represent an early event in gliomagenesis promoting a microenvironment conducive to tumor progression. To investigate this concept, we transiently expressed pp71 (rAD-pp71) or GFP (rAD-GFP) in adult neural precursor cells (NPCs –a glioma cell of origin model system) and measured changes in human oncogenes and tumor suppressor genes using a real-time PCR array. Multiple cellular gene products were specifically induced by pp71, especially the gene encoding stem cell factor (SCF/KITLG), with >50– fold induction (figure 2A). There was no induction of the gene encoding the SCF receptor, c-KIT; however several genes involved in growth and survival, cell cycle regulation, and DNA damage and apoptosis were upregulated by pp71 (figure 2A). We also observed a significant induction of the retinoblastoma gene (RB1), which is consistent with pp71’s ability to activate the E2F transcription factor [25].

To determine if the increased transcription of SCF correlated with production of the secreted SCF protein, we performed quantitative ELISA analysis using supernatant from NPCs, U87 glioma cells, and normal human astrocytes (NHA) infected with either rAD-pp71 or rAD-GFP. As shown in figure 2B, expression of pp71 resulted in a greater than two-fold induction of secreted SCF in both NPCs (p = 0.042) and glioma cells (p = 0.03), and a greater than four-fold induction in normal human astrocytes (p = 0.013), which have very low endogenous levels of SCF. This finding was confirmed by immunofluorescence in NPCs, where robust expression of SCF was observed in association with pp71 staining in HCMV-infected (TR) and pp71- transduced cells but not in the mock infected or GFP-transduced control cells (figure 2C).

To determine if pp71 expression is correlated to SCF expression in primary, endogenously infected GBM tumor samples, TaqMan analysis for SCF was performed on primary tissue samples previously analyzed for pp71 expression by TaqMan in figure 1B. Though SCF expression was variable, the average expression of SCF in pp71-positive GBMs was 2.4-fold higher than SCF expression in pp71-negative tumors (figure 2D). We also performed double immuofluorescence on passage zero cells cultured from primary GBMs to determine the localization of these proteins *in situ* (n = 5 primary cultures analyzed). Figure 2E shows a representative example, where pp71 and SCF protein expression are co-localized in a subset of primary GBM cells. As negative controls cells were stained with secondary antibody only or with anti-mouse and anti-rabbit isotype controls (figure S1C). Double immunofluorescence of primary GBM tissue sections for pp71 and SCF further demonstrates co-localization of the two proteins *in situ* (figure 2F). Negative controls (i.e., immunostaining of frozen tissue sections using secondary antibody alone) confirmed specificity of detection. The extent of pp71 and SCF co-localization was quantified in a small number of cells (n = 7) as described in [21], demonstrating that SCF was more highly expressed in pp71 positive GBM cells (Pearson co-efficient = 0.69, figure 2G). These data suggest a biologically relevant link between the presence of HCMV pp71 and SCF expression in human glioblastoma.

**Figure 3:**
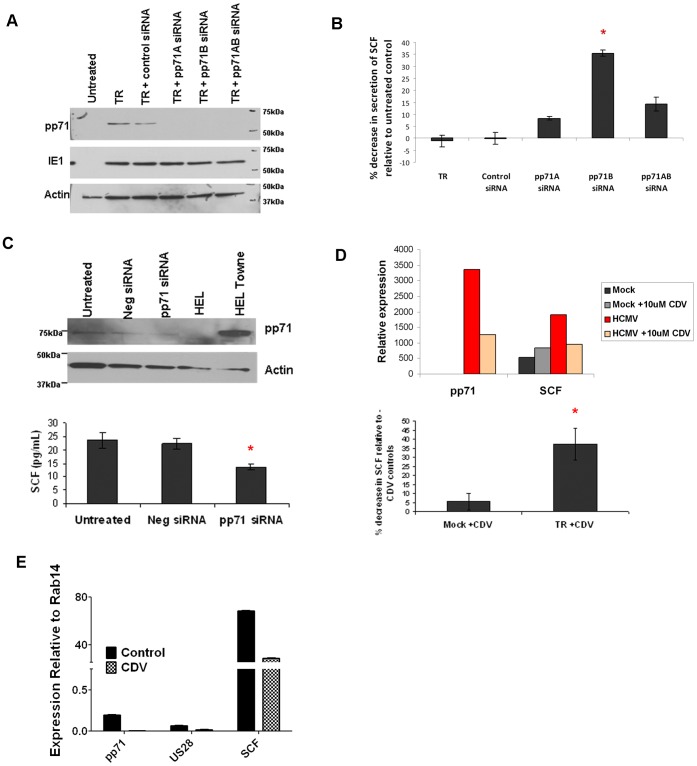
SCF induction by HCMV can be blocked by pp71 knockdown and Cidofovir. **A**: NPCs infected with HCMV (TR) were transfected with either negative control siRNA or a pp71-specific siRNA (designated A and B), pp71 protein levels were measured 72 hours later using western blot analysis (left panel). IE1 expression was determined to ensure specificity of the knockdown. **B**: Conditioned medium samples from the cells described in A were analyzed by ELISA for SCF. The percent decrease in SCF is displayed. (* p = 0.07 for pp71 B siRNA compared to negative control siRNA). **C**: HCMV positive, primary GBM cells transfected with control non-targeting siRNA or a siRNA specific to pp71 were analyzed by western blot for pp71 and actin 72h later. Percentage of pp71 knockdown (12.5%) from the western blot in C was calculated by normalization to actin (top panel). Conditioned medium from the same samples was analyzed by ELISA for secreted human SCF (bottom panel). *p = 0.003 by student t-test when pp71 siRNA was compared to negative control siRNA treatment. **D**: U87 cells mock or HCMV-infected were treated with 10uM cidofovir (CDV) or vehicle for 72 hours. RNA was collected and analyzed by TaqMan for pp71 and SCF and expression was normalized to Rab14 (top panel). Conditioned medium from the cells in E were analyzed by ELISA in triplicate. The percent decrease in SCF after CDV treatment compared to vehicle control is displayed (bottom panel). *p<0.05 as determined by a student t-test. **E**: Endogenously infected primary GBM cells (CPMC-145) were sorted for CD133 and the positive fraction was sub-cultured and treated with vehicle only or 10 uM CDV for 72 hours. RNA was collected and analyzed by TaqMan for pp71, US28 (another viral gene), SCF and Rab14.

To confirm that pp71 is specifically involved in the upregulation of SCF expression, we utilized RNA interference to knock down pp71 in both HCMV infected NPCs and a primary GBM culture. Using two siRNA sequences targeting pp71, we achieved a potent knockdown of pp71 protein expression in infected NPCs (figure 3A), which resulted in a decrease in SCF secretion as measured by ELISA (figure 3B). These siRNAs had no effect on the expression of another viral gene, IE1. While pp71 knockdown in an endogenously infected primary GBM culture was modest (12.5%) due to the required mild transfection conditions (figure 3C, top panels), we were able to detect a significant decrease in SCF secretion in the same cells by ELISA (p = 0.003, figure 3C, bottom panel). To test the effect of inhibiting pp71 expression on SCF expression using a clinically relevant paradigm, we infected U87 cells with HCMV and then treated the cells with the antiviral drug cidofovir (CDV), which is an acyclic nucleotide phosphonate, FDA-approved for treatment of HCMV infections. CDV did not have an effect on SCF expression in the absence of HCMV, however there was a 72.4% increase in SCF upon HCMV infection that was reduced by approximately 50% after CDV treatment (figure 3D, top panel). The amount of SCF secreted into the medium after CDV treatment of HCMV- infected cells (as measured by ELISA) was decreased by 37%, while CDV treatment of uninfected cells only led to a 5% decrease in SCF (p = 0.035, figure 3D bottom panel and figure S1D). As expected, pp71 expression was dramatically reduced (62%) after CDV treatment. CDV treatment of an endogenously infected GBM culture enriched for the CD133+ stem-like marker likewise suppressed expression of pp71 and SCF, as well as expression of HCMV US28 gene product, compared to vehicle-treated cultures (figure 3E). These results indicate that treatment with CDV can inhibit SCF expression in HCMV positive glioblastoma cells, suggesting a novel anti-tumor and anti-angiogenic therapeutic role for CDV.

While SCF expression levels positively correlate with glioma grade, SCF does not induce glioma cell proliferation despite the presence of SCF receptor, c-Kit, on tumor cells [5]. Therefore it has been proposed that SCF may be promoting glioma progression and angiogenesis through paracrine activation of c-Kit on endothelial cells present in the perivascular niche where GBMs develop [2], [5]. In order to determine if pp71- induced SCF can induce proliferation of NPC cultures, we performed a BrdU incorporation assay. NPCs that were untreated, infected with rAD-GFP, or incubated with rh-SCF did not show a significant increase in DNA synthesis (figure S2A). In contrast, pp71-transduced NPCs demonstrated an ∼8-fold increase in BrdU incorporation, which is likely due to pp71-mediated degradation of the hypophosphorylated form of Rb [25] (figure S2B).

**Figure 4:**
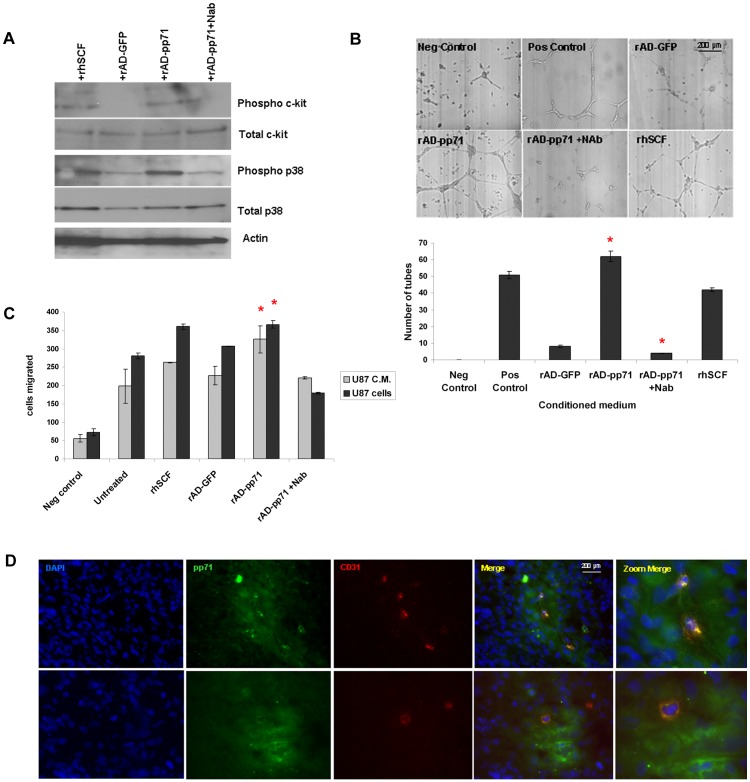
SCF induced by pp71 in NPCs and glioma cells is biologically active. **A**: HUVECs stimulated with either recombinant human SCF or conditioned medium from NPCs transduced with rAD-GFP, rAD-pp71, or rAD-pp71+ SCF neutralizing antibody were analyzed by western blot for phosphor-c-Kit, total c-kit, phosphor-p38, total p38, and actin. **B**: HUVECs were grown overnight in gel matrix supplemented as indicated. Capillary tubes that formed in each condition were visualized by microscopy (top panel), counted and plotted (bottom panel). *p = <0.05 as determined by student t-test when rAD-pp71 was compared to rAD-GFP and when rAD-pp71+Nab was compared to rAD-pp71. Each condition was run in duplicate and the experiment was repeated three times. **C**: HUVEC cells were subjected to a 4 h transwell migration assay using either 20,000 U87 cells treated as indicated (dark grey bars) or supernatant obtained from the same cells (light grey bars) on the bottom wells. For negative and positive control we used serum-free medium or rhSCF (1 ug/mL) respectively. HUVECs that migrated to the bottom of the well were stained and counted. *p = 0.015 for cells and 0.032 for supernatant when compared to untreated control, or 0.058 and 0.075 when compared to rAD-GFP, respectively by student t-test. Each condition was run in triplicate and the experiment was repeated twice. **D**: Frozen GBM tissue sections were immunostained for pp71 alone (figure 2F) or co-immunostained for pp71 (green) and the endothelial cell marker CD31 (red). Tissues were counterstained with DAPI. Two regions from a representative example from a GBM sample are shown. Magnification of the region of interest is shown in the last panel. Bar = 200µm. Negative controls can be found in figure 2F.

Since SCF-mediated c-Kit signaling can promote activation of the mitogen-activated protein kinase (MAPKs) and phosphoinositide 3-kinase (PI3K)/Akt pathways in target cells [31], we sought to determine if pp71-induced SCF could activate these downstream pathways in cultured human umbilical vein endothelial (HUVEC) cells. As shown in figure 4A, when HUVECS were stimulated with conditioned medium from NPCs transduced with pp71 (15 min), SCF-mediated phosphorylation of the c-Kit receptor was observed to a similar extent as that induced by authentic recombinant SCF ligand. This effect was specific, since c-Kit phosphorylation was not observed in HUVECs stimulated with conditioned medium from pp71-tranduced NPCs treated with a SCF neutralizing antibody. Consistent with the downstream signaling pathway for c-Kit, we also observed increased phosphorylation of p38-MAPK in cells treated with conditioned medium from pp71-expressing NPCs or recombinant SCF (Figure 4A).

Secreted SCF can directly activate brain microvascular endothelial cells (ECs) in vitro and induce a potent angiogenic response in glioblastoma in vivo [5]; therefore we hypothesized that pp71 expression could promote angiogenesis and used an in vitro endothelial tube formation assay to test this hypothesis. As shown in figure 4B, conditioned medium from NPCs that expressed pp71 induced a dramatic increase in capillary tube formation in HUVEC cells. Indeed, the extent of tube formation elicited by pp71 expression was comparable to that of the positive control media and to recombinant SCF. Overexpression of pp71 in U87 GBM cells also induced SCF secretion (Figure 2C), which promoted HUVEC tube formation (figure S2C). Taken together these data demonstrate that pp71-induced SCF derived from either NPCs or glioma cells is biologically active.

Both endothelial cell recruitment and capillary tube formation are essential features of glioma-induced angiogenesis, and since studies of the developing human central nervous system suggest that SCF may play a role in angioblast recruitment [32], we hypothesized that GBM cells that express pp71 may also increase endothelial migration via SCF secretion. As shown in figure 4C, untreated U87 cells or conditioned medium from these cells increased HUVEC transwell migration compared to the medium only negative control (which was expected, since U87 cells naturally secrete SCF). However, both pp71-expressing U87 cells and conditioned medium from these cells caused a significant increase in the transwell migration of HUVECs compared to control treated U87 cells (p = 0.015 and p = 0.032, respectively). HUVEC transwell migration induced by pp71 was comparable to that elicited by recombinant SCF, and was inhibited by pre-treatment with a SCF neutralizing antibody, demonstrating specificity. Overall, these data indicate that the SCF secreted by pp71-expressing GBM cells can directly promote endothelial cell migration.

Our data support a mechanism whereby pp71 expressed in GBM tumor cells, particularly the cancer stem-like cells, stimulates secretion of SCF in the tumor microenvironment, resulting in the activation and migration of nearby endothelial cells. To determine if pp71-expressing cells are proximal to endothelial cells in primary tumor specimens, we performed double immunofluorescence on frozen GBM tissue samples and found that pp71 positive GBM cells were indeed located adjacent to and surrounding the CD31 positive endothelial cells, further supporting our hypothesis for the pp71-induced paracrine signaling promoting GBM angiogenesis (figure 4D).

**Figure 5:**
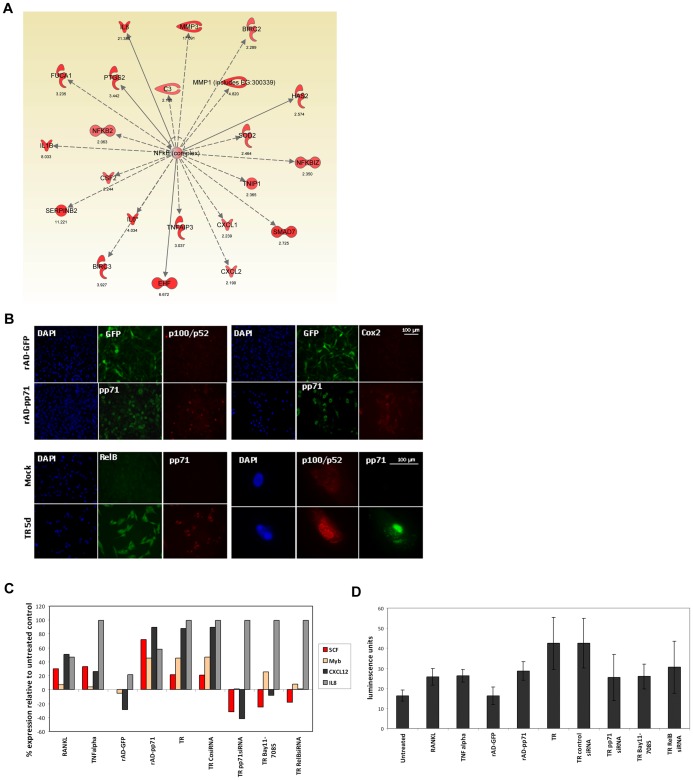
pp71 stimulation of SCF secretion requires activation of the NFKB signaling pathway. **A**: U87 cells stably transduced with a retrovirus encoding pp71 (pLXSN-pp71) or control (pLXSN) were used for transcriptome profiling using an Affymetrix Gene 1.0 ST array. Ingenuity pathway analysis (IPA) for differentially expressed genes identified several targets of the NFKB signaling pathway as being upregulated (fold-increase in expression is displayed next to the molecule icon). **B**: NPCs transduced with rAD-pp71, vector control, or HCMV- infected were co-immunostained for pp71 and markers of NFKB activation (p100/p52, Cox2, and RelB). Cells were counterstained with DAPI. **C**: Primary GSC neurospheres were treated as indicated and total RNA was analyzed by TaqMan for SCF, CXCL12, IL8, and Myb expression levels. Rab14 levels were used for normalization. The percent change in expression relative to untreated control is displayed. The experiment was repeated three times with similar results. **D**: U87 cells were transfected with an SCF-promoter driven luciferase construct and then treated as indicated. Luminescence readouts from a representative experiment are shown. Each condition was run in triplicate and the experiment was repeated three times. Positive (RPL13) and negative (scrambled) control promoter driven luciferase constructs were used. Differences were not statistically significant by student t-test.

To investigate the molecular mechanism underlying HCMV pp71 induction of SCF expression in glioblastoma, we generated a pp71-expressing stable U87 glioma cell line (figure S3A) and interrogated changes in gene expression between pp71- expressing and control cells using Affymetrix arrays, followed by Ingenuity pathway analysis (IPA) (raw Affymetrix data is available at the following link http://www.ncbi.nlm.nih.gov/geo/query/acc.cgi?token=hxutbkksiwaschc&acc=GSE42618). We found that NFΚB-stimulated genes were significantly upregulated in the pp71 expressing glioma cells (figure 5A). Among the 170 cancer-related molecules with altered expression, the top five activated regulators are all components of the NFΚB pathway (table 2). Since it has been reported that transcription of the SCF gene can be activated by NFΚB in a pro-inflammatory environment [33], we sought to investigate whether pp71 can activate NFKB signaling.

**Table 2:** U87-pp71 IPA pathway analysis summary.

Top Molecules	fold change		Top Biological Functions		
IL8	21.39		**Diseases and Disorders**	**p-value**	**# of molecules**
MMP3	17.09		Cancer	2.96E-15–4.34E-04	170
MMP12	15.06		Dermatological Diseases and Conditions	8.57E-12–4.23E-04	33
LIPH	14.36		Gastrointestinal Disease	2.62E-09–3.22E-04	89
FDCSP	11.69		Inflammatory Response	1.03E-08–4.10E-04	72
SERPINB2	8.20		Cardiovascular Disease	1.85E-08–2.44E-04	69
PI3	9.43				
SERPINB3	8.20		**Molecular and Cellular Functions**	**p-value**	**# of molecules**
IL1B	8.03		Cellular Movement	9.23E-09–4.34E-04	87
ZNF626	6.68		Cell Morphology	1.40E-09–4.34E-04	33
			Cellular Development	1.40E-08–4.34E-04	118
FABP3	−4.83		Cell-to-Cell Signaling and Interaction	2.14E-08–4.34E-04	64
RNU5A-1	−4.83		Cell Death and Survival	2.24E-08–3.88E-04	127
SCD	−4.52				
INSIG1	−3.91		**Top Upstream Regulators**		
HMGCS1	−3.40			**p-value**	**Outcome**
LDB2	−3.36		TNF	1.00E-20	Acivated
TMEM229B	−3.30		IL1B	8.07E-20	Acivated
NR2E1	−3.10		Lipopolysaccharide	3.77E-17	Acivated
PTP4A3	−2.92		IL1A	5.81E-16	Acivated
GABRG3	−2.90		RELA	1.73E-14	Acivated

Chart summarizing fold changes for the top 20 significantly altered genes in pp71 expressing versus control U87 cells (left panel). The top biological function alterations as determined by IPA software when compared to the Ingenuity knowledge base with associated p-values are listed (right panel).

To this end, we used NPCs either infected with whole virus (HCMV, TR clinical isolate) or transduced with rAD-pp71 and evaluated the status of NFKB pathway activation using immunostaining. We observed a mild activation of the canonical NFΚB pathway, as indicated by an increase in Cox2 staining and a robust activation of the non-canonical NFΚB component p100/p52 (NFKB2) in both pp71-transduced and HCMV-infected cells when compared to rAD-GFP or mock controls (figure 5B). The p52 heterodimer RelB was also induced in HCMV- infected cells and re-localized to the nucleus upon pp71 expression (figure S3B). It has been reported that RelB is upregulated by canonical NFKB signaling, which is known to be strongly activated by other viral genes [34], [35]. Our data demonstrate that pp71 was sufficient to activate processing of the inactive p100 protein to the active p52 form, as shown by its nuclear localization (for pathway diagram see figure S3C).

To corroborate nuclear re-localization with functional activation of the non-canonical NFKB pathway, we performed TaqMan for two NFKB-stimulated genes using primary GBM cells transduced with pp71. CXCL12 (SDF-1) gene expression is activated by the non-canonical NFKB signaling, while IL8 is a target of the canonical NFKB pathway. Treatment with the cytokines RANKL or TNFα were used as positive controls since both are known to activate NFKB signaling to varying degrees. Both CXCL12 and IL8 were moderately activated by RANKL and TNFα strongly induced IL8, indicating the NKFB signaling pathway is intact in this GBM cell line (figure 5C). Transduction with the control adenovirus (rAD-GFP) had a negligible effect on gene expression, while transduction with pp71 led to a robust increase in expression of CXCL12 and SCF and a modest increase in expression of IL8. To verify specificity of the pp71 effect on the NFKB signaling, we used Taqman analysis of HCMV-infected GBM cells treated with a combination of two siRNA oligonucleotides targeting pp71, or a scrambled, non-targeting siRNA. HCMV infection induced upregulation of SCF, CXCL12 and IL8, which was unaltered after transfection with the control siRNA. Upon treatment with pp71 siRNA, we measured a significant decrease in expression of SCF and CXCL12, indicating that pp71 is directly responsible for their activation. The effect on IL8 expression was modest, likely due to other viral genes which can stimulate canonical NFKB signaling and thus compensate for the loss of pp71.

Since pp71 only partially activates canonical NFKB signaling (known to activate the SCF promoter) we hypothesized that additional mechanisms underlying SCF stimulation exist. It has been reported the c-myb proto-oncogene binds the SCF promoter. Interestingly, our initial PCR-based screening shows 40-fold upregulation of c-myb after pp71 expression (figure 2A) [36]. Subsequent Taqman validation showed that c-myb expression was induced both by pp71 and HCMV, and that pp71 knockdown completely negated the whole virus-induced activation (figure 5C). RelB is essential for expression of the full-length c-myb transcript, therefore it is possible that NFKB and c-myb pathways are complementary mechanisms leading to SCF activation [37]. To confirm the role of NFKB in regulating SCF activation, we performed a promoter- based Luciferase reporter assay using U87 cells to measure activity of the SCF promoter (figure 5D). Luminescence measurements demonstrated a trend of SCF promoter activation upon stimulation with RANKL, TNFα, rAD-pp71 expression, or HCMV infection. pp71 knockdown or direct inhibition of NFKB using Bay11-7085 or RelB knockdown (figure S3D) reversed HCMV-induced activation of the SCF promoter. These results indicate that pp71 specifically upregulates SCF via activation of the non-canonical NFKB pathway.

**Figure 6:**
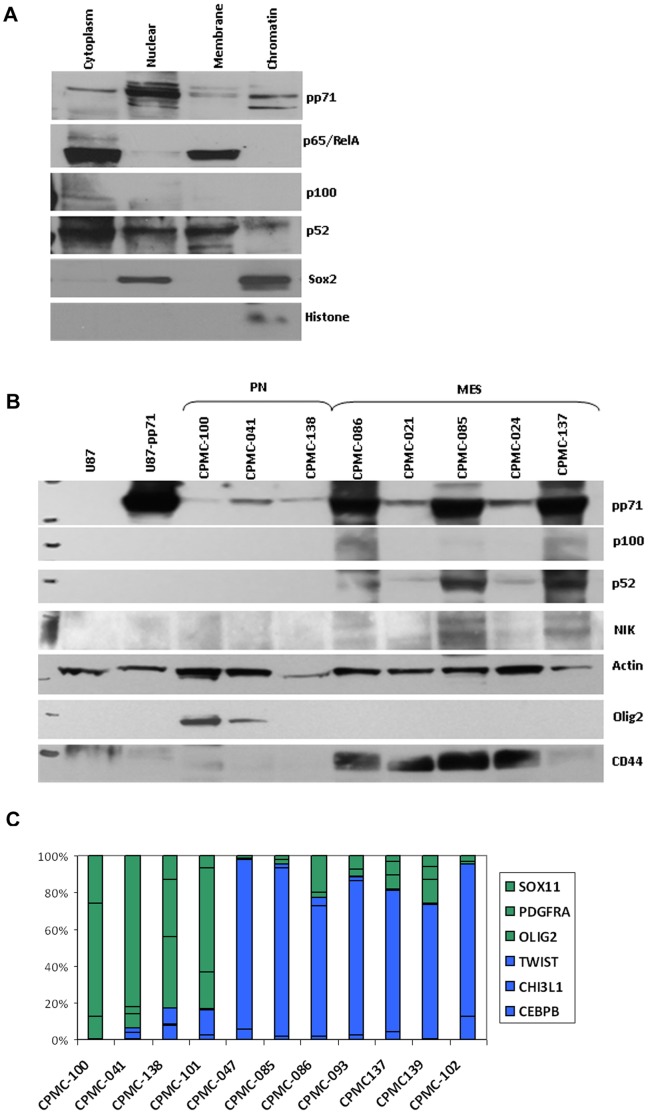
pp71 preferentially activates non-canonical NFKB signaling in endogenously infected glioblastoma tissues. **A**: Primary GBM tissue was processed using a subcellular protein fractionation kit. An equivalent amount of protein from each fraction was analyzed by western blot for pp71, p65/RelA, p100/p52, Sox2 and histone. **B**: Primary GBMs classified as proneural or mesenchymal using TaqMan analysis of differentially expressed transcripts (described in C) were analyzed by western blot for pp71, p100/p52, NIK, Actin, Olig2, and CD44. **C**: TaqMan was performed on GBM cDNA using probes to known mesenchymal (CEBP, CHI3L1, TWIST) and proneural (OLIG2, PDGFRα, Sox11) genes. The results show ΔΔCt for each marker relative to Rab14 in the tissues analyzed.

To validate the relevance of the NFKB signaling pathway in a primary GBM which endogenously expressed pp71, we subjected subcellular fractions from a homogenized primary GBM to western blot analysis. Figure 6A shows that pp71 is present in all cellular compartments, which supports our immunofluorescence data (Figure 2E, F) showing that pp71 expression is not strictly nuclear in primary GBM tumor cells. These data are not surprising, since GBM cells in situ do not exhibit the pattern of viral gene expression characteristic of a lytic infection. In this GBM sample, the canonical NFKB component p65/RelB appears largely inactive based on its localization in the cytoplasmic and membrane fractions; the p100 (inactive) subunit of the noncanonical pathway was detected only in the cytoplasm, indicative of clean fractionation (Figure 6A). However the activated p52 subunit was abundant in the nucleus as well as the chromatin, suggesting the noncanonical NFKB pathway is highly activated in this tumor. To corroborate this result in additional GBM specimens, we performed western blot analysis of whole cell lysates prepared from primary frozen GBM samples (figure 6B). pp71 was expressed in all tumor samples examined, but was much more abundant in tumors of the mesenchymal subclass, which are known to be more aggressive, invasive, and angiogenic (Figure 6B–C) [38], [39]. GBM sample classification was verified using Taqman for specific proneural and mesencymal markers and confirmed by western blot analysis for CD44 (mesenchymal marker) and Olig2 (proneural marker, Figure 6 B–C). The tumors with the highest expression of pp71 also displayed highest levels of expression of the activated noncanonical NFKB components p52 and NFKB inducing kinase (NIK), suggesting that pp71 expression may contribute to sustained activation of NFKB signaling in GBMs belonging to the mesenchymal molecular subtype.

## Discussion

Glioblastomas are characterized by a very high degree of vascular proliferation and angiogenesis, which promotes their aggressive growth and invasiveness [40]. Our group first reported that HCMV infection was present in a high percentage of GBMs [41]. Since HCMV encodes for multiple gene products that might enhance tumor aggressiveness, we have begun to examine these tumors for expression of viral gene products which may contribute to the complex GBM phenotypes.

Recent work by our group and others indicates that HCMV infection of human gliomas may promote mitogenic signaling, angiogenesis, invasion and other pathways that are mediated by HCMV gene products including IE1, gB, and US28 (reviewed in [42]). Given the potential oncomodulatory properties of the HCMV pp71 gene product [43], we sought to determine whether this viral gene was present in primary GBMs in situ and whether ectopic expression of pp71 in adult NPCs (the putative cells of origin of glioma) or glioma cells might contribute to the pathogenesis of this disease. Here, we demonstrate that HCMV pp71 RNA and protein are expressed in a majority of GBM specimens examined.

Our functional studies support a novel role for pp71 with important implications for glioma progression. We demonstrate that pp71 expression in adult NPCs and glioma cells induces SCF expression in a NFKB dependent manner. Importantly, SCF was recently shown to be a potent angiogenic factor in human GBM; furthermore, SCF expression levels are positively correlated with glioma grade and inversely correlated with patient survival [44]. Induction of SCF by pp71 in glioma precursor stem cells or transformed cells is likely to promote a pro-angiogenic microenvironment, which is critical for tumor progression.

We demonstrate here that pp71 expression in NPCs, U87 GBM cells, and normal human astrocytes increased secretion of biologically active SCF, capable of c-Kit receptor activation on endothelial cells. Knockdown of pp71 expression using siRNA or the anti-viral drug cidofovir in both *in vitro* infected cells and endogenously infected primary glioblastoma cells resulted in decreased SCF secretion, indicating a specific role for pp71 in driving SCF mediated pro-angiogenic signaling. Furthermore, conditioned media from pp71-expressing NPCs or U87 GBM cells induced endothelial tube formation and cell migration to levels to levels similar to those induced by recombinant SCF, and these activities were specifically blocked with a neutralizing antibody to SCF. Overall, these data strongly suggest that pp71 expression in human GBM cells in vivo may contribute to endothelial cell migration and angiogenesis, typically associated with an aggressive tumor phenotype.

Recent evidence has shown that a subpopulation of tumor cells, designated cancer stem cells, plays a critical role in GBM initiation and contributes to resistance to radiation and chemotherapy [45], [46]. This population of cells has also been demonstrated to express high levels of SCF in other solid tumors [47]. Importantly, we found pp71 preferentially expressed in the stem-like (CD133+) primary GBM cells in three cases we interrogated. Furthermore, immunostaining of frozen GBM sections revealed that pp71 is expressed in tumor cells surrounding the CD31 positive endothelial cells, an area of the tumor known to be enriched in glioma stem-like cells [48]. Of additional relevance to GBM stem cell survival is our finding that pp71 induces c-myb expression. c-myb is required for the maintenance and expansion of progenitor cells in the bone marrow and neurogenic regions of the adult brain [49]. Therefore, induction of c-myb by pp71 may contribute to the survival and expansion of glioma stem cells while also stimulating SCF secretion [49].

Using complementary approaches, we show that pp71 activated both the canonical and non-canonical NFKB pathways, leading to up-regulation of SCF as well as sustained overexpression of several pro-inflammatory cytokines (IL8, IL1B, IL6, LIF, PTGS2, IL1A), tissue remodeling matrix metalloproteinases (MMP3, 12, 1, 7), and angiopoietins, which are important for vasculogenesis (ANGPT1, ANGPTL4). These results (shown in table 2) suggest that persistent pp71 expression could contribute to glioma progression by activation of tumor cell survival pathways and increased invasion, in addition to pro-angiogenic signaling.

The findings we present here regarding the pro-angiogenic effects of pp71 in GBM may represent one component of a broader HCMV strategy in promoting angiogenesis in GBM. Recently, we and others have determined that HCMV US28 is expressed in human GBMs, and promotes tumor angiogenesis by upregulating vascular endothelial growth factor (VEGF) [19], [50], suggesting that US28 and pp71 may have complementary roles in driving pro-angiogenic signaling.

Taken together our studies describe a novel for HCMV pp71 as a tumor promoter and possible therapeutic target in glioblastoma. In addition, these data suggest that CMV-specific antiviral therapy could be a potentially efficacious approach to mitigate the oncomodulatory effects of HCMV in glioma patients.

## Supporting Information

Figure S1 **Detection of pp71 in primary GBM samples. A:** Sequence alignment of pp71 PCR products obtained from 8 different primary GBM specimen compared to 2 HCMV lab-adapted strains Ad169 and Towne. Sites where nucleotide changes were observed are underlined. The sites within pp71 that were mapped for Rb interaction (LXCXD motif) and DAXX interaction (hDAXX interaction domain) are displayed. **B**: pp71 and GAPDH TaqMan analysis of cDNA synthesized from fetal brain or 2 primary GBMs (CPMC101 and 102) using either the iScript or SSII cDNA synthesis kits. cDNA from the SSII kit was also diluted 1∶10 with water to alleviate the reported inhibitory effects of the cDNA synthesis components on downstream qPCR [51]. Bars represent the copy number of each transcript as determined by standard curve per ug of input RNA, therefore the effect of dilution is accounted for. **C**: Primary passage 0 GBM cells were fixed and immunostained with anti-mouse and anti-rabbit isotype control primary antibodies and counterstained with DAPI. **D**: Conditioned medium from U87 cells mock infected or infected with TR virus then treated with vehicle control or 10 uM cidofovir for 72 hours were collected and subject to ELISA for SCF in triplicate.
(TIF)

Figure S2 **SCF does not induce autocrine proliferation but does stimulate HUVEC tube formation.** **A:** NPCs were untreated, transduced with rAD-GFP or rAD-pp71 adenoviruses for 48 hours, or incubated with recombinant human SCF (1 ug/mL) for 24 hours in 0.1% serum and then labeled with BrdU for 60 minutes. Cells were then fixed, stained for BrdU, and counterstained with propidium iodide. The percentage of BrdU positive cells in each treatment group was calculated and plotted. (* p = 0.007 for rAD-pp71 compared to control adenovirus transduced cells). **B:** NPCs were mock treated or transduced with rAD-pp71 and were immunostained for total RB protein (green), pp71 (blue), and counterstained with propidium iodide (left panel). Cells lysates were also subjected to western blot analysis, where the faster migrating band represents the hypophosphorylated form of Rb (middle panel). Quantification of the two Rb bands was performed and normalized to actin (right panel). **C:** HUVECs were grown overnight in gel matrix and either negative control medium (serum and growth factor free), positive control complete medium, negative control medium plus recombinant SCF (+rhSCF, 1 ug/mL), or conditioned medium from U87 cells transduced with rAD-GFP, rAD-pp71, or rAD-pp71 followed by 1hour preincubation with neutralizing antibody to SCF. Capillary tubes that were formed in each condition were visualized by microscopy (left panel), counted and plotted (right panel).
(TIF)

Figure S3 **Modulation of NFKB signaling by pp71. A:** U87 cells were stably transduced with a pp71 expressing retrovirus (pLXSN-pp71) versus an empty vecor control (pLXSN) and pp71 expression was confirmed by immunostaining and western blot. **B:** NPCs were mock treated or transduced with rAD-pp71 and immunostained for RelB and pp71 and counterstained with propidium iodide. **C:** Ingenuity systems pathway analysis software was used to diagram components of both the canonical and non-canonical NFKB pathways predicted to be activated by pp71. **D:** U87 cells were tested for RelB expression by western blot with or without TNFα treatment to induce expression or after RelB siRNA treatment to knockdown expression. Actin was used as a loading control.
(TIF)

## References

[1] Rönnstrand L (2004) Signal transduction via the stem cell factor receptor/c-Kit. Cellular and Molecular Life Sciences 61: 2535–2548. 15526160 10.1007/s00018-004-4189-6 PMC11924424

[2] Matsui J, Wakabayashi T, Asada M, Yoshimatsu K, Okada M (2004) Stem Cell Factor/c-kit Signaling Promotes the Survival, Migration, and Capillary Tube Formation of Human Umbilical Vein Endothelial Cells. Journal of Biological Chemistry 279: 18600–18607. 14985355 10.1074/jbc.M311643200

[3] Purow B, Fine HA (2004) Antiangiogenic therapy for primary and metastatic brain tumors. Hematology/Oncology Clinics of North America 18: 1161–1181. 15474340 10.1016/j.hoc.2004.05.003

[4] Anderson JC, McFarland BC, Gladson CL (2008) New molecular targets in angiogenic vessels of glioblastoma tumours. Expert Reviews in Molecular Medicine 10: null-null. 10.1017/S1462399408000768 PMC2646508 18684337

[5] Sun L, Hui A-M, Su Q, Vortmeyer A, Kotliarov Y, et al (2006) Neuronal and glioma-derived stem cell factor induces angiogenesis within the brain. Cancer Cell 9: 287–300. 16616334 10.1016/j.ccr.2006.03.003

[6] Melnick M, Sedghizadeh PP, Allen CM, Jaskoll T (2012) Human cytomegalovirus and mucoepidermoid carcinoma of salivary glands: Cell-specific localization of active viral and oncogenic signaling proteins is confirmatory of a causal relationship. Experimental and Molecular Pathology 92: 118–125. 22101257 10.1016/j.yexmp.2011.10.011

[7] Mitchell DA, Xie W, Schmittling R, Learn C, Friedman A, et al (2008) Sensitive detection of human cytomegalovirus in tumors and peripheral blood of patients diagnosed with glioblastoma. Neuro Oncol 10: 10–18. 17951512 10.1215/15228517-2007-035 PMC2600830

[8] Zafiropoulos A, Tsentelierou E, Billiri K, Spandidos DA (2003) Human herpes viruses in non-melanoma skin cancers. Cancer Lett 198: 77–81. 12893433 10.1016/s0304-3835(03)00269-6

[9] Samanta M, Harkins L, Klemm K, Britt WJ, Cobbs CS (2003) High prevalence of human cytomegalovirus in prostatic intraepithelial neoplasia and prostatic carcinoma. J Urol 170: 998–1002. 12913758 10.1097/01.ju.0000080263.46164.97

[10] Harkins L, Volk AL, Samanta M, Mikolaenko I, Britt WJ, et al (2002) Specific localisation of human cytomegalovirus nucleic acids and proteins in human colorectal cancer. Lancet 360: 1557–1563. 12443594 10.1016/S0140-6736(02)11524-8

[11] Castillo JP, Yurochko AD, Kowalik TF (2000) Role of human cytomegalovirus immediate-early proteins in cell growth control. J Virol 74: 8028–8037. 10933712 10.1128/jvi.74.17.8028-8037.2000 PMC112335

[12] Cinatl J Jr, Vogel JU, Kotchetkov R, Wilhelm Doerr H (2004) Oncomodulatory signals by regulatory proteins encoded by human cytomegalovirus: a novel role for viral infection in tumor progression. FEMS Microbiol Rev 28: 59–77. 14975530 10.1016/j.femsre.2003.07.005

[13] Cobbs CS, Harkins L, Samanta M, Gillespie GY, Bharara S, et al (2002) Human cytomegalovirus infection and expression in human malignant glioma. Cancer Res 62: 3347–3350. 12067971

[14] Baryawno N, Rahbar A, Wolmer-Solberg N, Taher C, Odeberg J, et al (2011) Detection of human cytomegalovirus in medulloblastomas reveals a potential therapeutic target. The Journal of Clinical Investigation 121: 4043–4055. 21946257 10.1172/JCI57147 PMC3195466

[15] Price RL, Bingmer K, Harkins L, Iwenofu OH, Kwon C-H, et al.. (2012) Cytomegalovirus infection leads to pleomorphic rhabdomyosarcomas in Trp53+/− mice. Cancer Research. 10.1158/0008-5472.CAN-12-2425 PMC3500419 23002204

[16] Bhattacharjee B, Renzette N, Kowalik TF (2012) Genetic Analysis of Cytomegalovirus in Malignant Gliomas. Journal of Virology 86: 6815–6824. 22496213 10.1128/JVI.00015-12 PMC3393585

[17] Ranganathan P, Clark PA, Kuo JS, Salamat MS, Kalejta RF (2012) Significant Association of Multiple Human Cytomegalovirus Genomic Loci with Glioblastoma Multiforme Samples. Journal of Virology 86: 854–864. 22090104 10.1128/JVI.06097-11 PMC3255835

[18] Barami K (2010) Oncomodulatory mechanisms of human cytomegalovirus in gliomas. Journal of Clinical Neuroscience 17: 819–823. 20427188 10.1016/j.jocn.2009.10.040

[19] Slinger E, Maussang D, Schreiber A, Siderius M, Rahbar A, et al (2010) HCMV-Encoded Chemokine Receptor US28 Mediates Proliferative Signaling Through the IL-6-STAT3 Axis. Sci Signal 3: ra58. 20682912 10.1126/scisignal.2001180

[20] Soroceanu L, Akhavan A, Cobbs CS (2008) Platelet-derived growth factor-[agr] receptor activation is required for human cytomegalovirus infection. Nature 455: 391–395. 18701889 10.1038/nature07209

[21] Soroceanu L, Matlaf L, Bezrookove V, Harkins L, Martinez R, et al (2011) Human Cytomegalovirus US28 Found in Glioblastoma Promotes an Invasive and Angiogenic Phenotype. Cancer Research 71: 6643–6653. 21900396 10.1158/0008-5472.CAN-11-0744 PMC3206211

[22] Saffert RT, Kalejta RF (2007) Human Cytomegalovirus Gene Expression Is Silenced by Daxx-Mediated Intrinsic Immune Defense in Model Latent Infections Established In Vitro. J Virol 81: 9109–9120. 17596307 10.1128/JVI.00827-07 PMC1951389

[23] Hwang J, Kalejta RF (2007) Proteasome-dependent, ubiquitin-independent degradation of Daxx by the viral pp71 protein in human cytomegalovirus-infected cells. Virology 367: 334–338. 17590404 10.1016/j.virol.2007.05.037

[24] Lee H-R, Kim D-J, Lee J-M, Choi CY, Ahn B-Y, et al (2004) Ability of the Human Cytomegalovirus IE1 Protein To Modulate Sumoylation of PML Correlates with Its Functional Activities in Transcriptional Regulation and Infectivity in Cultured Fibroblast Cells. J Virol 78: 6527–6542. 15163746 10.1128/JVI.78.12.6527-6542.2004 PMC416510

[25] Kalejta RF, Bechtel JT, Shenk T (2003) Human Cytomegalovirus pp71 Stimulates Cell Cycle Progression by Inducing the Proteasome-Dependent Degradation of the Retinoblastoma Family of Tumor Suppressors. Mol Cell Biol 23: 1885–1895. 12612064 10.1128/MCB.23.6.1885-1895.2003 PMC149485

[26] Kalejta RF, Shenk T (2003) Proteasome-dependent, ubiquitin-independent degradation of the Rb family of tumor suppressors by the human cytomegalovirus pp71 protein. Proceedings of the National Academy of Sciences of the United States of America 100: 3263–3268. 12626766 10.1073/pnas.0538058100 PMC152280

[27] Trgovcich J, Cebulla C, Zimmerman P, Sedmak DD (2006) Human Cytomegalovirus Protein pp71 Disrupts Major Histocompatibility Complex Class I Cell Surface Expression. J Virol 80: 951–963. 16378997 10.1128/JVI.80.2.951-963.2006 PMC1346885

[28] Eyler Christine E, Wu Q, Yan K, MacSwords Jennifer M, Chandler-Militello D, et al (2011) Glioma Stem Cell Proliferation and Tumor Growth Are Promoted by Nitric Oxide Synthase-2. Cell 146: 53–66. 21729780 10.1016/j.cell.2011.06.006 PMC3144745

[29] Kalejta RF, Shenk T (2002) Manipulation of the cell cycle by human cytomegalovirus. Front Biosci 7: d295–306. 11779699 10.2741/kalejta

[30] Soroceanu L, Cobbs CS (2009) CNS Cancer: Models, Markers, Prognostic Factors, Targets, and Therapeutic Approaches; Van Meir E, editor: Humana Press (Springer).

[31] Jin K, Mao XO, Sun Y, Xie L, Greenberg DA (2002) Stem cell factor stimulates neurogenesis in vitro and in vivo. J Clin Invest 110: 311–319. 12163450 10.1172/JCI15251 PMC151087

[32] Hasegawa T, McLeod DS, Prow T, Merges C, Grebe R, et al (2008) Vascular precursors in developing human retina. Invest Ophthalmol Vis Sci 49: 2178–2192. 18436851 10.1167/iovs.07-0632 PMC4943084

[33] Reber L, Vermeulen L, Haegeman G, Frossard N (2009) Ser276 Phosphorylation of NF-kB p65 by MSK1 Controls SCF Expression in Inflammation. PLoS ONE 4: e4393. 19197368 10.1371/journal.pone.0004393 PMC2632887

[34] Yurochko AD, Kowalik TF, Huong SM, Huang ES (1995) Human cytomegalovirus upregulates NF-kappa B activity by transactivating the NF-kappa B p105/p50 and p65 promoters. Journal of Virology 69: 5391–5400. 7636984 10.1128/jvi.69.9.5391-5400.1995 PMC189383

[35] Wang X, Sonenshein GE (2005) Induction of the RelB NF-κB Subunit by the Cytomegalovirus IE1 Protein Is Mediated via Jun Kinase and c-Jun/Fra-2 AP-1 Complexes. Journal of Virology 79: 95–105. 15596805 10.1128/JVI.79.1.95-105.2005 PMC538727

[36] Sicurella C (2001) Defective Stem cell factor expression in c-myb null fetal liver stroma. Blood Cells Mol Dis 27: 470–478. 11259170 10.1006/bcmd.2001.0407

[37] Suhasini M, Pilz RB (1999) Transcriptional elongation of c-myb is regulated by NF-[kappa]B (p50/RelB). Oncogene 18: 7360–7369. 10602492 10.1038/sj.onc.1203158

[38] Phillips HS, Kharbanda S, Chen R, Forrest WF, Soriano RH, et al (2006) Molecular subclasses of high-grade glioma predict prognosis, delineate a pattern of disease progression, and resemble stages in neurogenesis. Cancer Cell 9: 157–173. 16530701 10.1016/j.ccr.2006.02.019

[39] Verhaak RGW, Hoadley KA, Purdom E, Wang V, Qi Y, et al (2010) Integrated Genomic Analysis Identifies Clinically Relevant Subtypes of Glioblastoma Characterized by Abnormalities in PDGFRA, IDH1, EGFR, and NF1. Cancer Cell 17: 98–110. 20129251 10.1016/j.ccr.2009.12.020 PMC2818769

[40] Prados MD, Levin V (2000) Biology and treatment of malignant glioma. Semin Oncol 27: 1–10. 10866344

[41] Cobbs CS, Harkins L, Samanta M, Gillespie GY, Bharara S, et al (2002) Human cytomegalovirus infection and expression in human malignant glioma. Cancer Res 62: 3347–3350. 12067971

[42] Soroceanu L, Cobbs CS (2011) Is HCMV a tumor promoter? Virus Research 157: 193–203. 21036194 10.1016/j.virusres.2010.10.026 PMC3082728

[43] Kalejta RF (2004) Human cytomegalovirus pp71: a new viral tool to probe the mechanisms of cell cycle progression and oncogenesis controlled by the retinoblastoma family of tumor suppressors. J Cell Biochem 93: 37–45. 15352160 10.1002/jcb.20177

[44] Sun L, Hui AM, Su Q, Vortmeyer A, Kotliarov Y, et al (2006) Neuronal and glioma-derived stem cell factor induces angiogenesis within the brain. Cancer Cell 9: 287–300. 16616334 10.1016/j.ccr.2006.03.003

[45] Park D, Rich J (2009) Biology of glioma cancer stem cells. Molecules and Cells 28: 7–12. 19655094 10.1007/s10059-009-0111-2

[46] Chen J, Li Y, Yu T-S, McKay RM, Burns DK, et al (2012) A restricted cell population propagates glioblastoma growth after chemotherapy. Nature 488: 522–526. 22854781 10.1038/nature11287 PMC3427400

[47] Levina V, Marrangoni AM, DeMarco R, Gorelik E, Lokshin AE (2008) Drug-Selected Human Lung Cancer Stem Cells: Cytokine Network, Tumorigenic and Metastatic Properties. PLoS ONE 3: e3077. 18728788 10.1371/journal.pone.0003077 PMC2518121

[48] Calabrese C, Poppleton H, Kocak M, Hogg TL, Fuller C, et al (2007) A Perivascular Niche for Brain Tumor Stem Cells. Cancer Cell 11: 69–82. 17222791 10.1016/j.ccr.2006.11.020

[49] Ramsay RG, Gonda TJ (2008) MYB function in normal and cancer cells. Nat Rev Cancer 8: 523–534. 18574464 10.1038/nrc2439

[50] Maussang D, Langemeijer E, Fitzsimons CP, Stigter-van Walsum M, Dijkman R, et al (2009) The human cytomegalovirus-encoded chemokine receptor US28 promotes angiogenesis and tumor formation via cyclooxygenase-2. Cancer Res 69: 2861–2869. 19318580 10.1158/0008-5472.CAN-08-2487

[51] Levesque-Sergerie J-P, Duquette M, Thibault C, Delbecchi L, Bissonnette N (2007) Detection limits of several commercial reverse transcriptase enzymes: impact on the low- and high-abundance transcript levels assessed by quantitative RT-PCR. BMC Molecular Biology 8: 93. 17953766 10.1186/1471-2199-8-93 PMC2151766

