# Supplementary figures and images for: Cytomegalovirus pp71 Protein Is Expressed in Human Glioblastoma and Promotes Pro-Angiogenic Signaling by Activation of Stem Cell Factor
Source: PLoS One. 2013 Jul 5;8(7):e68176. doi: 10.1371/journal.pone.0068176 (PMC3702580; doi:10.1371/journal.pone.0068176)

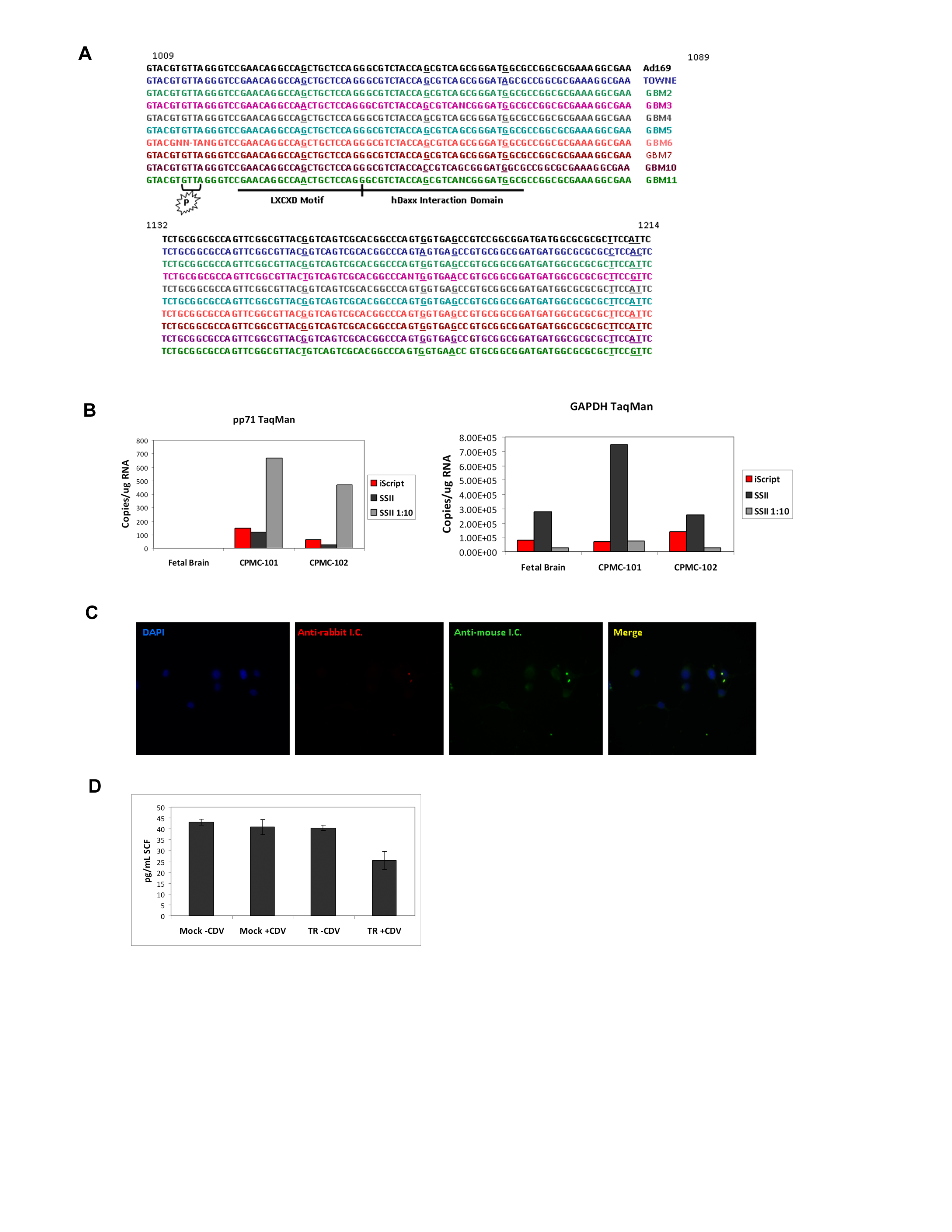

Supplement: Figure S1 — Detection of pp71 in primary GBM samples. A: Sequence alignment of pp71 PCR products obtained from 8 different primary GBM specimen compared to 2 HCMV lab-adapted strains Ad169 and Towne. Sites where nucleotide changes were observed are underlined. The sites within pp71 that were mapped for Rb interaction (LXCXD motif) and DAXX interaction (hDAXX interaction domain) are displayed. B: pp71 and GAPDH TaqMan analysis of cDNA synthesized from fetal brain or 2 primary GBMs (CPMC101 and 102) using either the iScript or SSII cDNA synthesis kits. cDNA from the SSII kit was also diluted 1∶10 with water to alleviate the reported inhibitory effects of the cDNA synthesis components on downstream qPCR [51]. Bars represent the copy number of each transcript as determined by standard curve per ug of input RNA, therefore the effect of dilution is accounted for. C: Primary passage 0 GBM cells were fixed and immunostained with anti-mouse and anti-rabbit isotype control primary antibodies and counterstained with DAPI. D: Conditioned medium from U87 cells mock infected or infected with TR virus then treated with vehicle control or 10 uM cidofovir for 72 hours were collected and subject to ELISA for SCF in triplicate. (TIF) [file pone.0068176.s001.tif]

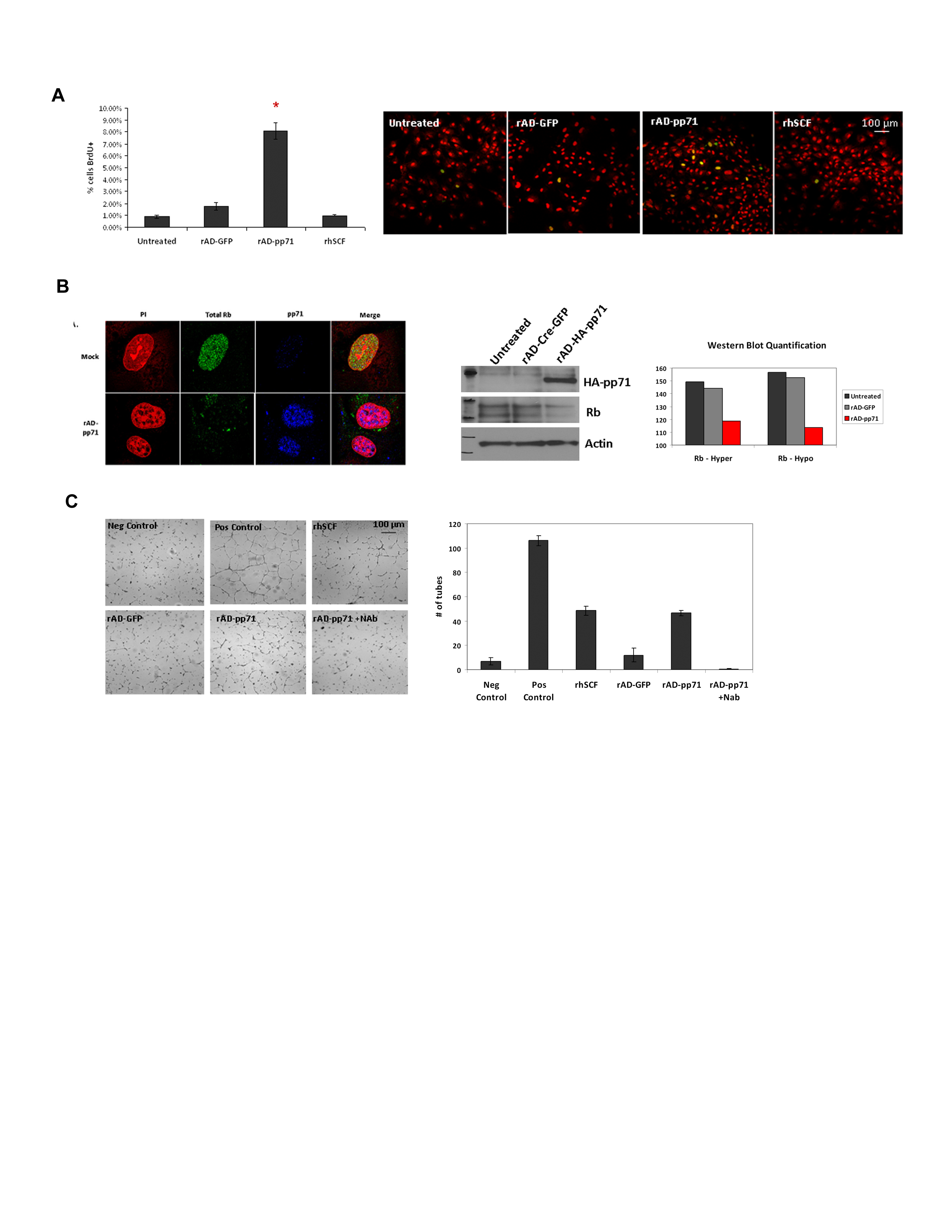

Supplement: Figure S2 — SCF does not induce autocrine proliferation but does stimulate HUVEC tube formation. A: NPCs were untreated, transduced with rAD-GFP or rAD-pp71 adenoviruses for 48 hours, or incubated with recombinant human SCF (1 ug/mL) for 24 hours in 0.1% serum and then labeled with BrdU for 60 minutes. Cells were then fixed, stained for BrdU, and counterstained with propidium iodide. The percentage of BrdU positive cells in each treatment group was calculated and plotted. (* p = 0.007 for rAD-pp71 compared to control adenovirus transduced cells). B: NPCs were mock treated or transduced with rAD-pp71 and were immunostained for total RB protein (green), pp71 (blue), and counterstained with propidium iodide (left panel). Cells lysates were also subjected to western blot analysis, where the faster migrating band represents the hypophosphorylated form of Rb (middle panel). Quantification of the two Rb bands was performed and normalized to actin (right panel). C: HUVECs were grown overnight in gel matrix and either negative control medium (serum and growth factor free), positive control complete medium, negative control medium plus recombinant SCF (+rhSCF, 1 ug/mL), or conditioned medium from U87 cells transduced with rAD-GFP, rAD-pp71, or rAD-pp71 followed by 1hour preincubation with neutralizing antibody to SCF. Capillary tubes that were formed in each condition were visualized by microscopy (left panel), counted and plotted (right panel). (TIF) [file pone.0068176.s002.tif]

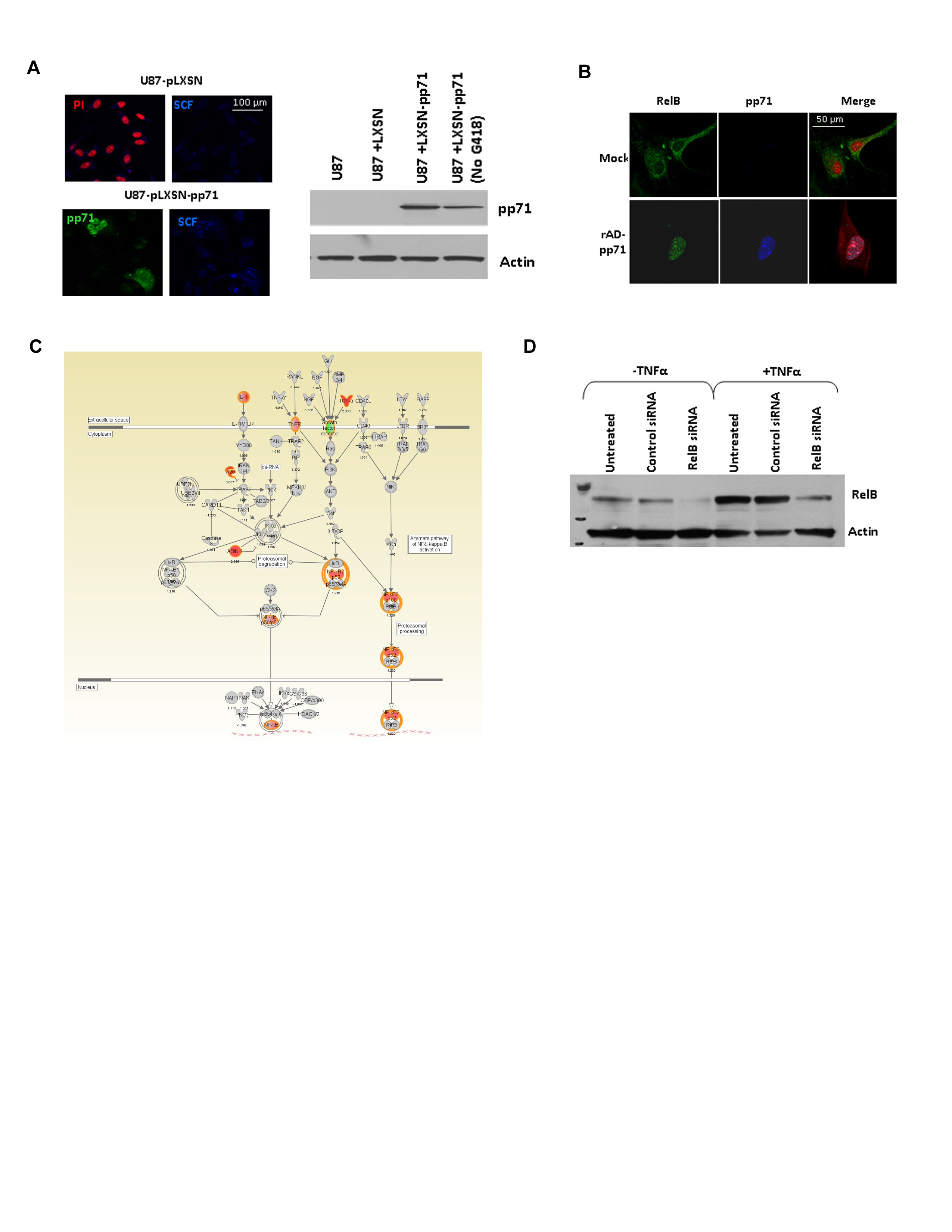

Supplement: Figure S3 — Modulation of NFKB signaling by pp71. A: U87 cells were stably transduced with a pp71 expressing retrovirus (pLXSN-pp71) versus an empty vecor control (pLXSN) and pp71 expression was confirmed by immunostaining and western blot. B: NPCs were mock treated or transduced with rAD-pp71 and immunostained for RelB and pp71 and counterstained with propidium iodide. C: Ingenuity systems pathway analysis software was used to diagram components of both the canonical and non-canonical NFKB pathways predicted to be activated by pp71. D: U87 cells were tested for RelB expression by western blot with or without TNFα treatment to induce expression or after RelB siRNA treatment to knockdown expression. Actin was used as a loading control. (TIF) [file pone.0068176.s003.tif]
